# Viral Infections and Schizophrenia: A Comprehensive Review

**DOI:** 10.3390/v15061345

**Published:** 2023-06-09

**Authors:** Ioanna Kotsiri, Panagiota Resta, Alexandros Spyrantis, Charalampos Panotopoulos, Dimitrios Chaniotis, Apostolos Beloukas, Emmanouil Magiorkinis

**Affiliations:** 1 Department of Internal Medicine, Asklipeion General Hospital, Voulas, 16673 Athens, Greece; 2 Department of Biomedical Sciences, University of West Attica, 12243 Athens, Greece; 3 National AIDS Reference Centre of Southern Greece, Department of Public Health Policy, University of West Attica, 11521 Athens, Greece; 4 Department of Clinical Cardiology, General Hospital of Kalamata, 24100 Kalamata, Greece; 5 Department of Laboratory Medicine, Sotiria General Hospital for Chest Diseases, 11527 Athens, Greece

**Keywords:** schizophrenia, congenital infections, prenatal viral infections, pregnancy and viral infections

## Abstract

Schizophrenia is a complex mental disorder with multiple genetic and environmental factors contributing to its pathogenesis. Viral infections have been suggested to be one of the environmental factors associated with the development of this disorder. We comprehensively review all relevant published literature focusing on the relationship between schizophrenia and various viral infections, such as influenza virus, herpes virus 1 and 2 (HSV-1 and HSV-2), cytomegalovirus (CMV), Epstein-Barr virus (EBV), retrovirus, coronavirus, and Borna virus. These viruses may interfere with the normal maturation of the brain directly or through immune-induced mediators, such as cytokines, leading to the onset of schizophrenia. Changes in the expression of critical genes and elevated levels of inflammatory cytokines have been linked to virally-induced infections and relevant immune activities in schizophrenia. Future research is necessary to understand this relationship better and provide insight into the molecular mechanisms underlying the pathophysiology of schizophrenia.

## 1. Introduction

### 1.1. The Disorder of Schizophrenia

Schizophrenia is a chronic mental disorder that affects approximately 1% of the world’s population. It is characterized by cognitive disturbances and diverse clinical manifestations, with symptoms usually emerging during puberty or early adulthood. These symptoms may include hallucinations, delusions, disorganized thinking, social withdrawal, and reduced emotional expression [1,2]. People with schizophrenia also commonly experience sleep and eating disorders and are at increased risk of co-morbidities, such as obesity, diabetes, arterial hypertension, and coronary disease [3].

The etiology of schizophrenia is believed to be multifactorial, involving both genetic and environmental factors. While genetic factors, such as mutant alleles and chromosomal abnormalities, may play a role in the onset of the disorder, its appearance among monozygotic twins at a rate of approximately 50% is considered strong evidence of environmental influence [1,4]. Environmental risk factors, including obstetric complications during labor, poor prenatal diet, prenatal exposure to viral infections, urban living, and smoking during adolescence, have been associated with fetal brain development and the subsequent development of neurodevelopmental and psychiatric disorders, including schizophrenia [5,6,7,8]. Individuals with a genetic predisposition, as described in the Neurodevelopmental Disorder Theory [1,4], may be more susceptible to the effects of these environmental factors on abnormal cerebral maturation [9,10,11,12,13,14]. The Neurodevelopmental Disorder Theory suggests that both genetic and environmental factors lead to abnormal cerebral maturation and that viral infections may contribute to the development of schizophrenia. The correlation between viral infections and schizophrenia is still controversial, with many published studies reporting conflicting results. In this review, we aim to summarize evidence on the relationship between viral infections and the onset of psychiatric disorders, including schizophrenia, in adulthood through a systematic search and critical review of published studies.

### 1.2. Pathophysiology of Schizophrenia

Understanding the pathophysiology of schizophrenia has been a significant challenge in the field of biology due to the lack of full elucidation of its causes. It is believed that schizophrenia and other psychotic disorders affect temporal structures, such as the hippocampus, which undergoes development during prenatal stages and many years before the onset of psychosis [11,14]. These disorders may impede the multiplication of neuronal cells and the formation of axial connections, leading to insufficient development of the central nervous system (CNS) [15]. Brain CT and MRI findings in patients have shown enlarged lateral cerebral ventricles and decreased volume of the cerebral cortex, mainly in the frontal and temporal lobes, as well as reduced hippocampal volume [15,16]. Brain CT and MRI findings in patients have shown enlarged lateral cerebral ventricles and decreased volume of the cerebral cortex, mainly in the frontal and temporal lobes, as well as reduced hippocampal volume. Apart from the anatomical changes in the brain, a recently published study proposed alterations in the regulation of genes associated with schizophrenia in various areas of the brain. Glavan et al. examined the expression of target genes in the amygdala, hippocampus, prefrontal cortex, and thalamus in post-mortem brain samples obtained from 20 suicide completers and 7 control subjects. Changes in expression of the GFAP (glial fibrillary acidic protein) gene, which is involved in the pathogenesis of schizophrenia, were observed. Moreover, the same study proposed novel molecular pathways that may affect the brain function of individuals with neurodevelopmental disorders [17].

#### Molecular Pathways in Schizophrenia

Research into the pathophysiology of schizophrenia has also focused on immune responses to viral or bacterial infections or immunogenic factors. Cytokines, which are systemic mediators, play an essential role in the host’s immune response to several infections. However, increased activity of the immune system may lead to the excessive release of inflammatory cytokines, which in turn may affect the growth of the central nervous system (CNS) [18,19]. As a family of soluble polypeptides, cytokines are considered robust biomarkers of infectious and inflammatory conditions [20].

The association between classical pro-inflammatory cytokines and schizophrenia has been examined, although the precise molecular mechanisms of this association remain unclear. The interaction between microglia and the complement system is considered a key player. For example, serum levels of interleukin-6 (IL-6) and tumor necrosis factor-α (TNF-α) are found to be raised in acute psychotic relapses in patients with schizophrenia. A meta-analysis conducted by Brian J. Miller et al. demonstrated increased levels of macrophage-derived cytokines IL-1, IL-6, and TNF-α, as well as the Th1-derived cytokines IFN-γ and IL-12, in patients with established schizophrenia [21,22].

Irregular IL-6 concentrations have been found to be responsible for neurodevelopmental abnormalities in many cases in the literature, and there are a variety of proposed molecular mechanisms that mediate these events [23]. Therefore, it is believed that IL-6 may interfere in the kynurenine pathway, in which many Toll-like receptors (TLRs), such as TLRs 2, 4, 8, and 9, are involved [22]. Tryptophan metabolism can be shifted into the kynurenine pathway, resulting in kynurenic acid production, due to the activation of indoleamine-2,3-deoxygenase (IDO1). IL-6 was found to be responsible for the IDO1 induction. Kynurenic acid is an antagonist of the N-methyl D-aspartate (NMDA) receptor in the CNS [22]. It is now well known that NMDA receptors play a crucial role in a wide variety of brain functions, and many NMDAR disruptions are found to be responsible for neurodegenerative events [24].

In a study by S. Giovanoli et al., the consequences of prenatal immune activation in mice were examined in relation to latent neuropsychiatric disorders. Prenatal infection was found to “prime” the developing organism’s sensitivity to subsequent environmental challenges postnatally. The study measured the levels of the inflammatory molecules interleukin-1b (IL-1b), tumor necrosis factor-a (TNF-a), and prostaglandin E2 (PGE2) in two different brain areas, the hippocampus (HPC) and prefrontal cortex (PFC), after prenatal immune activation and peripubertal stress. The results showed elevated levels of the proinflammatory cytokines IL-1b and TNF-a in hippocampal microglia responses [25].

**Figure 1:**
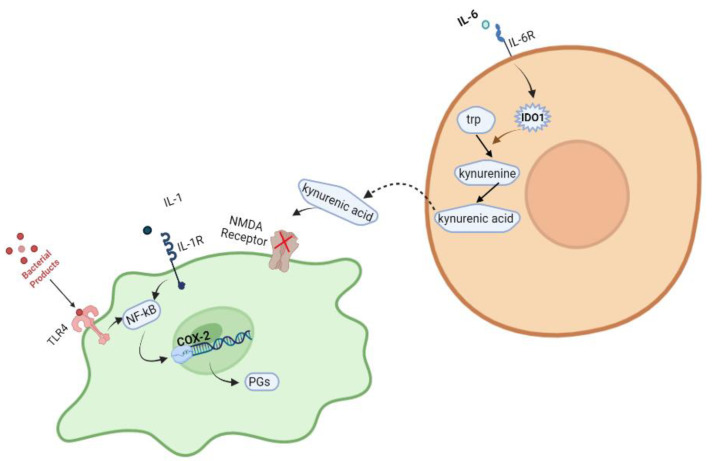
Various intracellular pathways have been reported to be associated with the development of schizophrenia. Created with BioRender.com: https://www.biorender.com/ accessed on 28 February 2023.

Another molecular pathway implicated in the pathophysiology of schizophrenia involves overexpression of the COX-2 gene in nerve cells. The COX enzyme is responsible for the production of signaling molecules called prostaglandins (PGs), which play important roles in neuronal activity, cell apoptosis, and several other physiological processes in the central nervous system. The pro-inflammatory cytokine IL-1 binds to the IL-1 receptor, and a complex signaling network is induced. This network, in turn, activates a cascade of events mediated by the transcription factor NF-κB, ultimately leading to the activation of the COX-2 gene [26,27,28]. Additionally, various Toll-like receptors appear to initiate molecular pathways that result in COX-2 gene expression. TLRs trigger the innate immune response by detecting conserved molecular patterns for early immune recognition of a pathogen. For example, TLR4 binds to bacterial products and stimulates an intracellular signaling pathway in which NF-κB is one of the downstream transcription factors to be activated, ultimately increasing the expression of the COX-2 gene once again. These molecular pathways are briefly described in Figure 1.

Another molecular mechanism that has been examined in terms of the pathophysiology of schizophrenia is a dysfunction in the neurotransmitter system, mainly the dopamine system. This can result in increased production and activity in the limbic system of the brains of individuals with schizophrenia. Cellular stress, as well as blocking dopaminergic neurons, can lead to increased serotonin secretion. Meanwhile, the glutamine neurotransmitter exhibits reduced activity in the prefrontal cortex of the patient’s brain, where genes encoding regulators of presynaptic function display changes in expression. Specifically, a number of single nucleotide polymorphisms (SNPs) have been identified in the regulator of G-protein signaling 4 (RGS4) gene, and some of these SNPs have been identified as having transmission bias [29,30,31]. However, further experimental evidence is needed.

It is important to investigate the risk factors that trigger these physiological and molecular changes, as they are already considered possible therapeutic targets. Treatment of schizophrenia with a wide variety of anti-inflammatory and immunomodulatory agents has been shown to be beneficial. Selective cyclooxygenase-2 (COX-2) inhibitors, such as celecoxib and rofecoxib, have already been tested as promising therapeutic options [32,33,34].

We emphasize the putative association of these molecular pathways with the development of schizophrenia and the potential for targeted therapies to alleviate symptoms and improve patient outcomes.

## 2. Risk Factors

Several risk factors for schizophrenia have been reported in the literature, with environmental factors and genetic predisposition being the most significant contributors to its development. Pregnancy complications have also been identified as potential risk factors, while viral infections may indirectly affect critical molecular pathways with neurodegenerative consequences and could be considered as possible risk factors in the manifestation of schizophrenia. This review focuses on the association between certain viral infections and schizophrenia; however, it is crucial to consider additional factors and explore further aspects to gain a comprehensive understanding of schizophrenia’s manifestation.

### 2.1. Environmental Risk Factors

Idemiological studies, as well as studies on monozygotic twins, have shown that a wide variety of environmental risk factors can affect the premature development of the brain [35]. In their studies, Hare et al. and Machon et al. reported that children who were born at the end of the winter season or early spring showed an increased incidence of schizophrenia [36,37]. These findings correspond with other studies that reported an increased rate of schizophrenia occurrence by 5–15% in childbirths during these months, which could additionally be related to viral infection outbreaks during the winter and early spring seasons [38,39,40]. Moreover, urban childbirth and the upbringing of children are considered environmental risk factors in the manifestation of adult schizophrenia. This correlation could also be explained by the increased exposure to infectious agents in densely populated areas, toxic exposures, social deprivation and fragmentation, income inequality, etc. A very large cohort of 2.66 million Danish people was studied by C. B. Pedersen and P. B. Mortensen to assess environmental risk factors that might be linked to the development of schizophrenia. Overall, the urbanity of birthplace was underlined as a possible risk factor in the group of 10,264 persons who developed schizophrenia during the 50.7 million person-years of follow-up [41,42].

### 2.2. Pregnancy Complications

Intensely stressful situations during pregnancy have been additionally associated with an increased risk of developing psychiatric disorders in the offspring [32]. The Northern Finland Birth Cohort 1966 (NFBC 1966), a large cohort study, included 10,934 individuals who had been living in Finland since their mothers’ mid-pregnancy. Of these individuals, 150 (1.4%) had developed schizophrenia. Published literature highlights possible risk factors, including complications during pregnancy. For instance, perinatal brain damage was found to be a risk factor for developing schizophrenia, with an odds ratio (OR) of 4.6. Low (OR 2.5; 95% CI 1.2–5.1) and high (OR 2.4; 95% CI 1.1–4.9) birth weights, as well as low (OR 2.6; 95% CI 1.1–5.9) and high (OR 1.8; 95% CI 1.0–3.5) birth lengths, were also considered risk factors [43]. Maternal stress during the gestational period may cause placental vasoconstriction and delayed intrauterine growth of the fetus [44]. The disturbance of the hypothalamic-pituitary-adrenal axis is thought to be responsible. Various nutrient deficiencies, especially a lack of folic acid, which is essential for cerebral development, may lead to an increased risk of neurodevelopmental disorders [45]. Complications during gestation, labor, and delivery may increase the risk of schizophrenia in offspring, as several studies have shown [40,46]. Case-control studies have shown a high rate of schizophrenia in adults with complications during childbirth, especially in those with genetic vulnerability [46,47,48,49]. This could be due to the hypoxia of the fetus, which leads to irreversible injury of the hippocampus and the amygdale [34,46]. Schizophrenia has also been linked with congenital malformations, a small circumference of the head, suffocation during childbirth, uterine atony, perinatal bleeding, and ischemic injuries [50,51,52].

Once again, high levels of pro-inflammatory cytokines during pregnancy have been associated with neurodegenerative events [53,54]. Specifically, increased cytokine levels during gestation, such as TNF-a, IL-1b, and IL-6, may adversely affect the development of the fetal brain as they cross the placenta, which may be synthesized by the mother, the placenta itself, or the fetus. The levels of these maternal cytokines have been shown to be elevated in human pregnancies in which the offspring exhibit schizophrenia, both in prenatal and postnatal infections. Overall, the risk of schizophrenia is increased by the molecular events of the immune response, such as inflammatory cytokines and discs, instead of the specific pathogen itself [35,35,55,56,57,58].

### 2.3. Viral Infections

**Figure 2:**
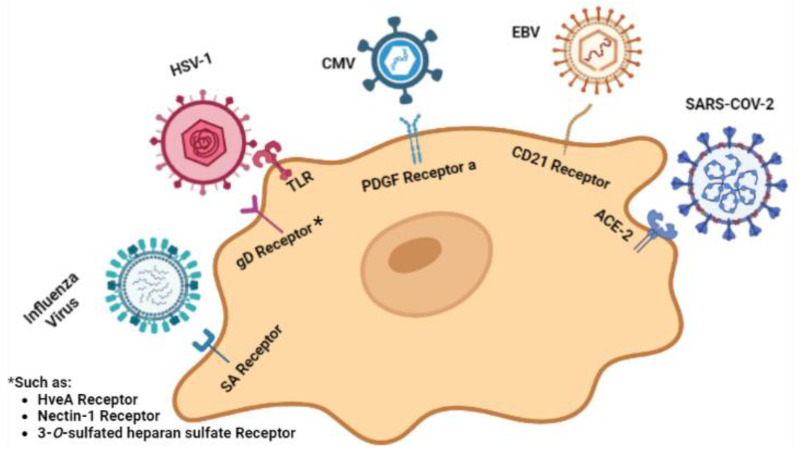
Viruses targeting neuron cells and their relevant cellular receptors. Created with BioRender.com: https://www.biorender.com/ accessed on 28 February 2023.

Psychiatric disorders may occasionally be triggered by certain viral infections, although the exact molecular mechanisms are not yet fully understood. Several studies have shown that maternal infections during the first and second trimesters of pregnancy can lead to psychological disorders and potentially contribute to the development of psychiatric illnesses in offspring during adolescence or adulthood [59,60,61]. To understand the underlying molecular pathways, most studies have focused on neuroinvasive and neurotropic viruses, along with their neurotropic receptors on a typical neuron cell, which are illustrated in Figure 2 [62].

#### 2.3.1. Influenza Virus

The influenza virus is a member of the Orthomyxoviridae family. A viral infection causes a human disease with symptoms including a high fever, cough, body ache, and runny nose. The viral genome consists of single-stranded RNA in the negative sense, is composed of eight segments, and most of the time infects epithelial cells [63]. Influenza virus infection during pregnancy has been identified as a risk factor for the appearance of schizophrenia in offspring during adulthood [40]. As previously mentioned, there are various studies showing that most people with schizophrenia are born during the winter months and early spring, when flu outbreaks occur. There is much discussion as to whether the direct offspring infection leads to neurodevelopmental disorders, which ultimately lead to schizophrenia, or whether it is due to the production of maternal cytokines in response to the infection [13,37,38]. Animal models examining the first case have shown that the influenza A virus may have accessed the CNS through either the olfactory route or the vagus nerve. Subsequently, it can affect some critical brain centers, such as the hippocampus, through the deregulation of neurotransmission [64]. I. Mori et al. tested this hypothesis in a transient infection model with the influenza A virus strain WSN/33 targeted to brain regions known for neuropsychiatric disturbances. Their results showed that in some specific thalamic areas, infection was followed by an almost total loss of neurons within 12 days [65]. Another study, conducted by Beraki et al., examining a transient, nonlethal influenza A virus infection in the brain, demonstrated that alterations in the regulation of some genes related to schizophrenia, such as the RGS4 gene, can cause persistent behavior changes [29]. The translational regulation of the myxovirus (influenza virus) resistance 2 (Mx2) gene has also been examined in MAM-treated rats, and the results showed a downregulation in the mRNA expression. Mx2 mRNA levels were found to be significantly decreased in both first-episode, drug-free, and chronically medicated schizophrenia patients [66]. Lastly, when it comes to the indirect causes of neurodevelopment disorders, there is much discussion about the increased levels of maternal pro-inflammatory cytokines, specifically interleukin IL-8, tumor necrosis factor (TNF)-a, IL-6, and C-reactive protein, and whether they are associated with a higher risk of psychosis in the offspring [67].

Many epidemiological studies have been conducted to examine the relationship between influenza virus infection and schizophrenia. Mednick et al. investigated the possibility of psychiatric disorders in adults who were infected with the influenza virus as fetuses. They included 1781 patients diagnosed with schizophrenia who were born during the 1957 flu A2 epidemic in Helsinki. The study showed an increased risk of schizophrenia among individuals whose second trimester of fetal life coincided with the flu epidemic, compared to those whose fetal life coincided with the first or third trimester [68]. Limosin et al. conducted another study comparing 973 schizophrenic patients with an equal number of non-schizophrenic patients and their non-schizophrenic brothers. They found an association between exposure to the influenza virus and the manifestation of schizophrenia. The number of schizophrenic patients who were exposed to the flu virus, especially during the second trimester of pregnancy, was much higher than the number of controls [69]. O’Callaghan et al. reported an 88% increase in schizophrenic adults born during the February to mid-March period in England and Wales. Their study included 616 patients born between 1983 and 1988 and diagnosed with schizophrenia. They divided them into two groups: patients with a family history of psychiatric disorders and patients without a family history. The study showed a significant increase only in those who had no family history of any psychiatric disorder, supporting the view that environmental factors are responsible for the occurrence of this disorder [70].

Moreover, Brown et al. used serological methods to document prenatal exposure to influenza and the increased risk of schizophrenia. Their study included 64 patients diagnosed with schizophrenia and 125 controls, all born between 1959 and 1966. Maternal serum samples were tested for influenza antibodies to estimate the prevalence in the population during the same period. The results showed that the risk of schizophrenia was increased in people exposed intrauterinely to the flu virus during the first trimester of pregnancy compared to those in the second or third trimester [71]. On the other hand, Torrey et al. examined birth data from 43,778 people with schizophrenia and compared it to 10,496,686 births between 1950 and 1959 in ten US states. They concluded that there was no significant increase in the schizophrenic birth rate just before, during, or after the 1957 flu epidemic [72]. In conclusion, whether the influenza virus is associated with schizophrenia or not is still a controversial subject in the published literature.

#### 2.3.2. Herpesviridae Family

Herpesviridae is a family of DNA viruses that infect humans and other animals initially through the mucosal surface and are also capable of migrating to the sensory ganglia [73,74]. There are eight (or nine, depending on the classification) herpesviruses for which humans are the primary host. Herpesviruses are not totally eradicated after their initial infection but remain dormant, are incorporated into the host genome, and are reactivated during immunosuppression.

Exposure to herpes viruses is examined as a possible risk factor for developing schizophrenia. Some members of the Herpesviridae family that are associated with schizophrenia are herpes simplex viruses (HSV) 1 and 2, cytomegalovirus (CMV), Epstein-Barr virus (EBV), human herpes virus 6 (HHV-6), and varicella-zoster virus (VZV) [75].

##### Herpes Simplex Viruses (HSV)

Herpes simplex viruses (HSV) are DNA viruses transmitted through nasal or oral secretions that, for the most part, cause mild or no symptoms at all. The most serious complication caused by the virus is encephalitis with severe morbidity, which can lead to psychosis and cognitive impairment [76]. Herpes simplex virus (HSV) has a tropism for neurons, is capable of replicating in the brain, and, for that reason, has been implicated in schizophrenia. As mentioned earlier, the immune response plays a crucial role in the development of these diseases. An association between increased levels of antibodies against HSV-1 and reduced levels of cognitive functioning has been observed in individuals with schizophrenia [77].

A mechanism that could explain the correlation between HSV-1 infection and activation of microglia in the brain involves Toll-like receptors (Figure 2). TLRs are receptors with extracellular leucine-rich repeats and an intracellular signaling Toll/Interleukin-1 (IL-1) receptor domain that are able to sense specific molecular patterns. TLR2, TLR3, and TLR9 are responsible for recognizing HSV-1, and all of them are expressed in microglia. Various ILs, type I IFNs, and tumor necrosis factor (TNF), inflammatory cytokines that have already been analyzed for their correlation with schizophrenia, are being expressed after TLR2 signals [77].

Several studies have reported an association between HSV-1 infection and cognitive impairment in schizophrenia. However, some of these studies found no significant association between HSV-2, HSV-6, and VZV infections and schizophrenia [78,79,80]. Nevertheless, Mohagheghi et al. conducted a study in which they measured IgM and IgG serum titers against HSV-1, HSV-2, and CMV in 45 patients with schizophrenia and 45 controls. The results showed that anti-CMV, anti-HSV1, and anti-HSV2 IgG antibodies were significantly higher in patients with schizophrenia compared to controls [81]. Mortensen et al. also investigated the possible correlation between maternal exposure to HSV-2 and the risk of schizophrenia in their adult offspring. They examined the blood serum of 602 schizophrenic patients and 602 controls and found an increased risk of schizophrenia in offspring whose mothers tested positive for serum HSV-2 IgG [82]. However, a study conducted by Thomas et al. examined whether intrauterine exposure to HSV-1 is associated with an increased risk of developing schizophrenia and reduced cognitive function. The study included 171 patients with schizophrenia, 27 with schizophreniform disorders, and 100 controls, all of whom were evaluated for exposure to HSV-1 using serum HSV-1 antibody titers. The results did not show a significant relationship between exposure to HSV-1 and the risk of schizophrenia. Lastly, a study conducted by Brown et al. investigated whether maternal exposure to HSV-2 was associated with the risk of schizophrenia in adulthood. Sixty prenatal serum samples were examined, and the authors found no correlation between prenatal exposure to HSV-2 and the risk of schizophrenia [64]. These controversial findings highlight the need for further investigation into the relationship between HSV and neurodevelopmental disorders.

##### Cytomegalovirus (CMV)

Cytomegalovirus (CMV) can be transmitted both vertically, from mother to fetus, and horizontally, through close personal contact. CMV is found in the saliva, blood, urine, vaginal fluids, and breast milk of infected individuals. It has been implicated as a source of inflammation in inflammatory bowel diseases, such as ulcerative colitis [83]. CMV infection is commonly found among individuals with low socioeconomic status [84]. Maternal infection or reactivation during pregnancy leads to congenital infection of the fetus. Congenital CMV infection is characterized by hepatosplenomegaly, jaundice, microcephaly, congenital mental retardation, hearing and vision loss, and cerebral calcifications [85,86]. CMV is considered the most common cause of damage to the fetal nervous system, and, therefore, it is believed that there may be a causal link between congenital infection and schizophrenia [87].

Lucchese et al. created a list of 26 different heptapeptides that are shared between HCMV and human proteins related to neuronal migration in their study. These human proteins hold the key to many processes occurring in the brain, from fetal-early postnatal neurodevelopment to adult neurogenesis. A large overlap of viral peptides with human proteins associated with neuronal migration has been observed. It is proposed that disturbances in GABAergic and glutamatergic circuitry may potentially lead to psychotic syndromes [88].

In Hoffmann et al.’s study, a possible reduction in brain volume in CMV-infected embryos has been investigated using MRI. The study included 27 CMV-infected embryos and 52 uninfected embryos. Infected fetuses had significantly lower brain volumes than controls. This, combined with a genetic predisposition, may increase the risk of developing schizophrenia later in life [89].

An increased risk of schizophrenia has also been implied because of the association between variations of tumor necrosis factor-α and interleukin-10 genes and susceptibility in both CMV infections and schizophrenia [90]. Moreover, complement C4 genes are loci related to schizophrenia susceptibility that are activated in response to infections. Saverance et al. found that in patients with schizophrenia, CMV IgG levels were inversely correlated with C4S copy numbers (R2 = 0.13–0.16, *p* < 0.0001) [83]. Additionally, increased levels of IgM antibodies against CMV were also found in the CSF. In this case, scientists were not able to determine whether it was a primary infection or a reactivation of the virus [91]. Similar results were presented by Dickerson et al., who studied 323 individuals with schizophrenia in whom elevated CMV antibody titers were found [92]. Albrecht et al. also reported elevated CMV antibody titers in 19 of 60 schizophrenic patients’ cerebrospinal fluid [93]. On the other hand, there are a number of published studies that examine serum antibody titers against CMV in patients with schizophrenia where no significant differences were observed between them and the controls [44,94,95].

##### Epstein-Barr Virus (EBV)

Epstein-Barr virus (EBV) is another virus belonging to the Herpesviridae family that can cause persistent infection and is associated with immunomodulatory effects. EBV is capable of infecting the central nervous system, and although it commonly results in short-duration fever and lymphadenopathy, it can also be asymptomatic (Figure 2). Exposure to EBV has also been associated with attachment anxiety and psychotic experiences in adolescence [96].

It is possible that an early-life infection could cause an exaggerated response in microglia to subsequent infections, resulting in adverse consequences for neuronal survival and functioning [97]. Recently, new data have provided evidence of the causal link between the Epstein-Barr virus and the development of multiple sclerosis (MS), underscoring the neurodegenerative action of EBV [98]. In Dickerson et al.’s study, serum IgG EBV antibodies were measured in 432 people with schizophrenia and 311 controls. Patients with schizophrenia had elevated levels of antibodies to EBV viruses compared to the control population [99]. It is becoming clear that future studies might need to focus on the putative association between EBV and schizophrenia in order to shed light on more aspects of this subject.

#### 2.3.3. Retroviruses

Retroviruses are enveloped RNA viruses that cause immunodeficiency and neurological disease in humans. A typical retrovirus genome consists of the gag-pol-env genes [100]. With the help of a reverse transcriptase, retroviruses can convert their RNA into DNA, and this retroviral DNA can be incorporated into the cellular DNA. Humans can be infected by the human immunodeficiency virus (HIV) and the human T-cell leukemia virus. These viruses can replicate their genome within the central nervous system and cause neurological and psychiatric symptoms in some infected individuals. Retroviruses exist as exogenous or endogenous entities. Exogenous retroviruses infect cells through a specific receptor, while endogenous retroviruses are present in the genome of host cells and are inherited through successive generations [101].

HIV infection has been found to be responsible for some well-characterized psychotic diseases, such as major depressive disorder and anxiety disorder. Additionally, HIV-associated neurocognitive disorders (HAND) are common among HIV patients, but there is not much evidence in the literature regarding HIV and schizophrenia [100].

Human endogenous retroviruses (HERVs), which belong to the human family of endogenous W-type retroviruses (HERV-W), have attracted the attention of the scientific community in the last decade. Several studies have since investigated whether there is a causal link between HERVs and schizophrenia, with the HERV-W family showing the greatest correlational indications [102].

In their study, Huang et al. collected blood samples from 58 individuals with schizophrenia and 38 controls. Using RT-PCR, they examined the RNA levels of the HERV pol gene in their blood. Retroviral pol genes were found to be elevated in 20 out of 58 subjects with schizophrenia. Additionally, elevated antibody titers against the ERV9 pol protein were observed in the serum of patients with schizophrenia but not in controls. As a result, Huang et al. concluded that the activation of certain retroviral proteins of the HERV-W family in some patients can lead to schizophrenia [103].

In a different study, scientists tried to detect genes similar to the human endogenous gene HERV-W env with mRNA sequencing in the plasma of 118 subjects with recent onset of schizophrenia and 106 physiological controls. The authors detected the target gene in 42 out of 118 individuals with schizophrenia, but none in the control group participants of the study. Using human glioma cells U251, they found that HERV-W env overexpression regulates a number of molecular markers associated with schizophrenia, such as the neurotrophic factor derived from the brain (BDNF), the N2-receptor receptor type of neurotrophic tyrosine kinase type 2 (NTRK2, also known as TrkB), and dopamine receptor D3, and increases cAMP phosphorylation. They also showed that HERV-W env activates BDNF production in human U251 glial cells. Overall, HERV transcriptional activation is associated with the development of schizophrenia in some patients, and HERV-W env regulates the expression of schizophrenia-related genes [104].

Furthermore, Yolken and colleagues investigated HERV-W RNA expression in the frontal cortex of postmortem brains from four individuals with schizophrenia, four with bipolar disorder, and six controls using RT-PCR. They found that HERV-W expression was significantly increased in subjects with schizophrenia compared to controls [105]. In a separate study, Perron et al. used an immunoassay with monoclonal antibodies to quantify HERV-W gag and envelope proteins in the serum of 49 subjects with schizophrenia and 49 healthy individuals. They found a positive antigenemia for envelope protein in 47% of patients and for gag antigen in 49% of patients, compared to only 3% for env and 4% for gag in healthy subjects. Additionally, a significant correlation was found between gag or env antigenemia and C-reactive protein, indicating inflammation as a possible cause of pathophysiology [106].

Karlsson and colleagues identified similar nucleotide sequences in retroviral pol genes in cerebrospinal fluid (CSF) in 10 of 35 patients (29%) diagnosed with schizophrenia and schizophreniform disorders with recent onset, as well as in the CSF of one out of 20 subjects with chronic schizophrenia. These retroviral sequences, mainly related to the HERV-W family, were not detected in any of the 32 healthy participants. In the same study, brain tissue was obtained postmortem from the frontal cortex of five subjects with schizophrenia and six individuals with no psychiatric history. They found increased RNA transcription levels of HERV-W genes in the brain tissue of individuals with schizophrenia compared to controls [107].

Yao et al. reported elevated transcription levels of HERV-W gag in mononuclear blood cells obtained from 30 patients with psychosis, including schizophrenia, schizophreniform disorder, and schizoaffective disorder, compared to 26 healthy controls. However, there was no difference in HERV-W env transcription levels [108]. Overall, these studies support the indirect contribution of HERV to schizophrenia, emphasizing the need for further research.

#### 2.3.4. Borna Virus

Borna virus (BDV) is a negative-sense single-stranded RNA virus that belongs to the Bornaviridae family and naturally infects horses, sheep, poultry, and cattle. As a neurotropic virus, BDV infects the brain and causes nerve cell degeneration in the chronic phase of the disease [109]. BDV infection of the CNS in animals can cause sporadic neurological diseases, such as encephalitis, meningitis, various abnormalities in movement and behavior, and sometimes psychological manifestations similar to schizophrenia [110]. In a study by M.V. Ovanesov et al., activated astrocytes by BDV infection were found to stimulate microglia that acquire a round shape, express MHC I, MHC II, and IL-6, and display increased secretion of TNF-α and IL-1 [111]. These cytokines have already been discussed as possible molecular biomarkers for the occurrence of schizophrenia. Several studies have focused on whether BDV disease can directly cause psychiatric disorders in humans, such as schizophrenia and depression. Nevertheless, the majority of these studies have shown that BDV infection does not cause psychiatric manifestations in humans directly, especially schizophrenia, even though BDV is an infectious agent discussed in many cases of depression. We refer to several epidemiological studies on this subject.

Yong et al. examined in their study whether BDV infection may, in some cases, be associated with schizophrenia. They measured BDV p24 antibodies in the sera of 116 schizophrenic patients and control subjects, and the results showed that infection is possibly associated with schizophrenia [112]. The study of Iwata et al., however, showed the opposite results. BDV p24 RNA was measured in monocytes of the peripheral blood (PBMCs) of psychiatric patients, 49 of whom had mood disorders, 77 had schizophrenia, and 84 were control individuals. The results did not show a greater prevalence of BDV p24 RNA in patients with psychiatric illness compared to the control group. Similar results were found by the study of Selten et al., who showed that BDV infection does not play any role in the pathogenesis of schizophrenia [113,114]. The same negative correlation was shown by a study conducted in Iran, where the possibility of BDV infection-causing psychiatric disorders was considered. They examined samples of peripheral blood for the detection of BDV RNA nucleoprotein P40 from 120 patients (60 with bipolar disorder and 60 with schizophrenia) and 75 controls. The results did not show any correlation between BDV infection and the pathogenesis of these psychiatric disorders [115]. Lastly, an examination of the correlation between BDV infection and schizophrenia was conducted in a study that included 40 individuals with the first onset of schizophrenia and 40 controls. The researchers measured BDV RNA in PBMCs isolated from patients with the first onset of schizophrenia. The results showed that there was no causal link between the Borna virus and schizophrenia [116].

#### 2.3.5. Coronaviruses (CoVs)

Coronaviruses (CoVs) are the largest RNA viruses identified [117]. Viruses from three genera can infect humans. Their genome encodes four structural proteins, namely the spike (S), nucleocapsid (N), membrane (M), and envelope (E) [117]. The COVID-19 pandemic has been declared a global health emergency by the World Health Organization (WHO), and in addition to psychological trauma, patients with COVID-19 may experience psychotic symptoms, such as delusion, disorganized speech, confusion, seizure, stroke, and disorganized behaviors [118].

While the novel human coronavirus SARS-CoV-2 is mostly known for its tropism towards lung cells, it has also been detected in the trachea, small intestine, kidney, and brain. The ACE-2 (angiotensin-converting enzyme 2) receptor appears to be responsible for SARS-CoV-2 cell invasion (Figure 2) [119].

The neurotropism of the virus was examined in human induced pluripotent stem cell (hiPSC)-derived monolayer brain cells and region-specific brain organoids by Zimmer et al. [102]. Their study revealed infection of neural cells within 6 h of incubation with SARS-CoV-2 and replication of the virus within the cells at 72 h. Several mechanisms have been proposed for SARS-CoV-2 neuroinvasion, including direct infection of neural tissue by the ACE-2 receptor.

A well-characterized event, the cytokine storm, has triggered scientific interest in the cases of COVID-19 patients. Although the blood-brain barrier (BBB) in the CNS is an anatomical border, the increased production of cytokines can affect its function, causing BBB breakdown and, eventually, brain damage. Several increased pro-inflammatory and anti-inflammatory mediators in the cytokine storm are also common in other viral infections known to increase the risk of neurodevelopmental disorders. The main components of the cytokine storm are interleukin 6 (IL-6) and C-reactive proteins. Having already mentioned the possible role of IL-6 in schizophrenia, a number of different molecular markers associated with neuropsychiatric disorders triggered by SARS-CoV-2 infection are listed in Table 1 [120].

In 2011, Severance et al. conducted a study on 106 patients with early-onset psychotic symptoms and 196 controls and observed a higher seropositivity for HKU1, NL63, and OC43 coronaviruses [127]. Zambrano et al. proposed that COVID-19 is linked to low birth weight infants and an increased risk of preterm deliveries, but this remains a controversial topic [128]. Lokken et al. performed a small cohort study on women infected with SARS-CoV-2 during late pregnancy and found that known risk factors for COVID-19 severity in the general population can increase the need for a premature C-section to alleviate respiratory difficulty [129]. Following COVID-19 diagnosis in pregnant patients, fetal growth restrictions [130], low birth weight of babies [131], miscarriages, and stillbirths have also been reported [130,132]. A proposed thesis based on animal models suggests that higher levels of maternal choline might mitigate some effects of infection on fetal brain development [133]. However, many cohort studies claim that there is no increased risk of premature delivery or impaired fetal growth triggered by SARS-CoV-2 infection.

#### 2.3.6. Other Viral Infections

Some epidemiology studies suggest that parvovirus B19 infection may play a role in the development of schizophrenia, but further research is needed to confirm this in larger studies [134,135]. In 1992, Eagles proposed a potential link between poliovirus infection and schizophrenia, but this hypothesis was not explored further [136,137]. There has also been a hypothesis that Zika virus infection may contribute to the development of schizophrenia, but there is no clinical evidence supporting this theory [138,139]. However, as a neurotropic virus, Zika’s impact on brain development has been widely researched, and it is now known that Zika infection in pregnant women can lead to microcephaly in their offspring [140]. Since the human brain continues to develop into early adulthood, viral infections during this period, particularly those that affect the nervous system, may increase the risk of various neurodevelopmental outcomes [97,141].

## 3. Discussion

Schizophrenia is a complex, long-term mental disorder that appears to be the outcome of both heritable and developmental-environmental risk factors. Some environmental contributors are birth complications, nutrient deficiency, medications during pregnancy, maternal stress during gestation, urbanization, and viral infections. This last factor is the main subject of this review. Prenatal exposure, especially in the first trimester of gestation, is crucial for the brain development of the fetus. Histologic abnormalities in brain centers, such as the hippocampus, influence an individual’s likelihood of developing a psychotic disorder [20].

A possible mechanism has been proposed that includes many pro-inflammatory cytokines, explaining the causal link between viral infection and schizophrenia. These modulators can act either directly on neurons or through neurotransmission. Boin et al. mentioned in their study that tumor necrosis factor α (TNFα), a well-characterized cytokine, can exert neurotoxic effects on cell growth and proliferation depending on its concentration. This observation can be explained after taking into consideration the fact that the TNFα gene is located at a locus (6p21.1–21.3) that has already been associated with genetic susceptibility to schizophrenia. Moreover, the complement system, a well-known part of the innate and adapted immune response to viral infections, has been examined by Albert C. and Yang et al., among others. Increased complement protein activity, especially C1, C3, and C4, was found to contribute to the accelerated pruning of synapses. On the same topic, Presumey et al. mentioned in their study that the complement system plays a significant role in the mature brain, stimulating synapse loss in the early stages of neurodegenerative diseases [142,143,144].

Neurodevelopmental disorders can possibly be triggered after a maternal viral infection with pathogens, such as the Zika virus, cytomegalovirus, rubella, and herpes simplex virus. It has already been mentioned that, among other genetic and/or environmental factors, maternal pro-inflammatory cytokines can play a part in the abnormal maturation of neuron cells. In the infrequent case in which a pathogen crosses the trophoblast, an unfavorable environment in the fetus’ brain can be formed. A well-characterized example is HSV-1. This neurotropic pathogen is known for its significant role in neurodevelopmental disabilities in children who were exposed prenatally. Furthermore, due to its high vertical transmission risk, CMV, another member of the Herpesviridae family, became, in the last decade, a prognostic factor for sensorineural loss and neuropsychological disorders, such as schizophrenia, in infants [145].

## 4. Conclusions

Experimental and epidemiological studies suggest a possible link between viral infections and the development of schizophrenia. Some viral infections can disrupt the normal development of the fetus’s central nervous system by activating the maternal immune system. Alterations in the expression of crucial genes and increased levels of inflammatory cytokines are believed to be the connecting link between viral infections and schizophrenia. While it is clear that various factors, such as pathogens, complications, and medications during pregnancy can contribute to the risk of developing neurodevelopmental disabilities, including schizophrenia, more research is necessary to understand the molecular mechanisms and virally induced pathophysiology of this disorder.

## Figures and Tables

**Table 1:** Alterations in genes’ regulation in schizophrenia.

Symbol	Full Name	Biological Functions	Alteration in Gene Regulation in Schizophrenia	Related Study
APOL2	Apolipoprotein L2	Transport of lipids and binding of lipids to organelles.	The APOL 2 and 4 genes, located on chromosome 22q12.3–q13.1, are known to be upregulated in the brains of schizophrenic patients.	Lee et al., 2013 [121]
APOL4	Apolipoprotein L4	Exchange and transport of lipids/cholesterol.		Lee et al., 2013 [121]
CHI3L1	Chitinase 3 like 1	Glycosyl hydrolase is associated with inflammation and tissue remodeling.	Gene expression studies indicate that CHI3L1 mRNA levels are increased in the hippocampus and dorsolateral prefrontal cortex of postmortem samples with schizophrenia compared with control samples.	Yang et al., 2008 [122]
MTHFR	Methylenetetrahydrofolate reductase	Conversion of 5,10-methylenetetrahydrofolate to 5-methyltetrahydrofolate.	MTHFR contributes to the regulation of RELN,56 whose expression is decreased in the hippocampus of subjects suffering from schizophrenia and major depressive disorder. It is possible that the low activity of MTHFR combined with other genetic and environmental factors contributes to the developmental outcome.	Favre et al., 2012 [123] and Kezurer et al., 2013 [124]
SETD1A	SET domain containing 1A, histone lysine methyltransferase	Histone methyltransferase complex.	Loss-of-function (LOF) variants that lead to disruption of SERD1A are likely to contribute to the etiology of neuropsychiatric disorders.	Takata et al., 2014 [125]
SYN2	Synapsin II	Neuronal phosphoproteins associated with synaptogenesis and neurotransmitter release.	Decreased levels of synapsin II have been reported in schizophrenia.	Egbujo et al., 2015 [126]

## Data Availability

Not applicable.
